# A Prospective Review on the Research Progress of Citric Acid Modified Starch

**DOI:** 10.3390/foods12030458

**Published:** 2023-01-18

**Authors:** Ming Zhang, Hongyu Jia, Bin Wang, Chao Ma, Fatao He, Qi Fan, Wei Liu

**Affiliations:** 1 Jinan Fruit Research Institute All China Federation of Supply and Marketing Co-Operatives, Jinan 250014, China; 2 Shandong Academy of Agricultural Sciences Institute of Agricultural Resources and Environment, Jinan 250132, China; 3 Key Laboratory of Agro-Products Processing, Institute of Food Science and Technology, Chinese Academy of Agricultural Sciences, Ministry of Agriculture and Rural Affairs, Beijing 100193, China

**Keywords:** citric acid, starch, hydrolysis, esterification, structure, function

## Abstract

Citric acid (CA) treatment is a convenient, mild and environmentally friendly strategy to modify the composition, structure and function of starch through hydrolysis and esterification, which expands the application of starch in industry. In this paper, the effects of CA modification on amylose content, amylopectin chain length distribution, microscopic morphology, solubility and swelling ability, thermodynamic properties, gelatinization properties, digestibility properties, texture properties and the film-forming properties of starch were summarized. The application status and development trend of CA modified starch were reviewed, which has important implications for the targeted utilization of CA modified starch in the future.

## 1. Introduction

Starch is a polysaccharide composed of glucose units and is the main storage form of carbohydrates in higher plants [1]. Starch is rich in natural resources, which has a good biodegradability and biocompatibility, and is widely used in biopharmaceuticals, food, health products, cosmetics, textiles, papermaking, environmental protection and other fields [2]. However, the inherent properties of native starch limit its industrial application, including: easy aging, insolubility in cold water, low shear resistance, increased consistency after thermal gelatinization, and poor thermal stability [3]. Through chemical, physical, enzymatic hydrolysis [4], genetics [5] and other methods, the physical and chemical properties of starch can be changed, achieving a modified starch with special properties which can be used in more scenarios.

**Figure 1:**
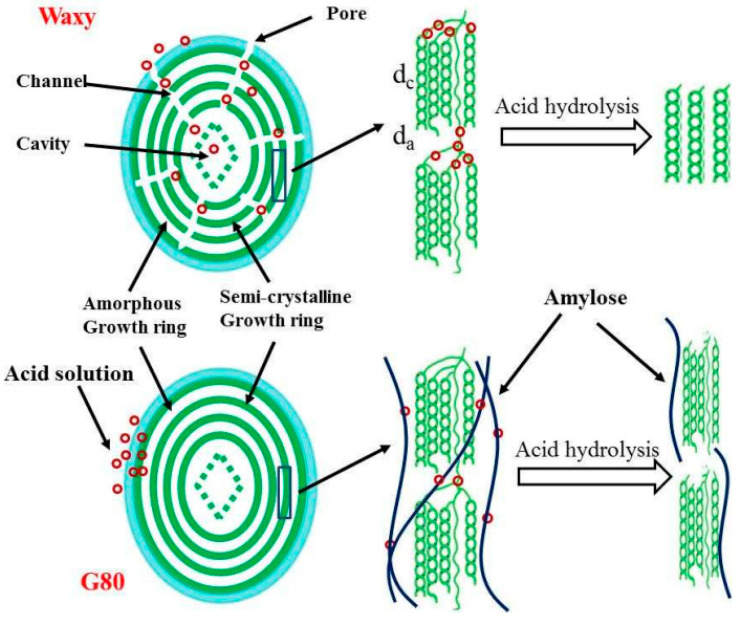
The mechanism of the acid hydrolysis of starch “Reprinted/adapted with permission from Ref. [6]. Copyright © 2017, Elsevier”.

Acid hydrolysis and esterification are two common chemical methods in the industrial production of modified starch. The process of acid hydrolysis is relatively simple. The starch is suspended in an organic acid or inorganic acid solution, followed by stirring, filtering, neutralizing, washing and drying steps (the temperature in the process is always lower than the starch gelatinization temperature). The starch retains the granular structure after acid hydrolysis, while its viscosity decreases and fluidity increases. As shown in Figure 1, the mode of action of acid hydrolysis is related to the composition and structure of starch. The surface of waxy starch granules contains micropores, and acid molecules enter the interior of the starch granules from the micropores, forming channels in an “inside-out” manner. Hydrolysis is carried out, while the surface structure of high amylose granules is relatively complete and smooth, and the acid molecules are hydrolyzed in a “from outside to inside” manner [6]. The alcoholic hydroxyl groups of starch molecules are esterified by inorganic and organic acids to obtain esterified modified starch. The esterification reaction can reduce starch aging, dehydration and condensation, and improve the transparency, gloss, emulsion stability and thermal stability of starch paste, and film formation. In industrial production, inorganic acids, such as hydrochloric acid and sulfuric acid, are generally used to modify starch, but the acid-containing waste liquid has adverse effects on the environment.

**Figure 2:**
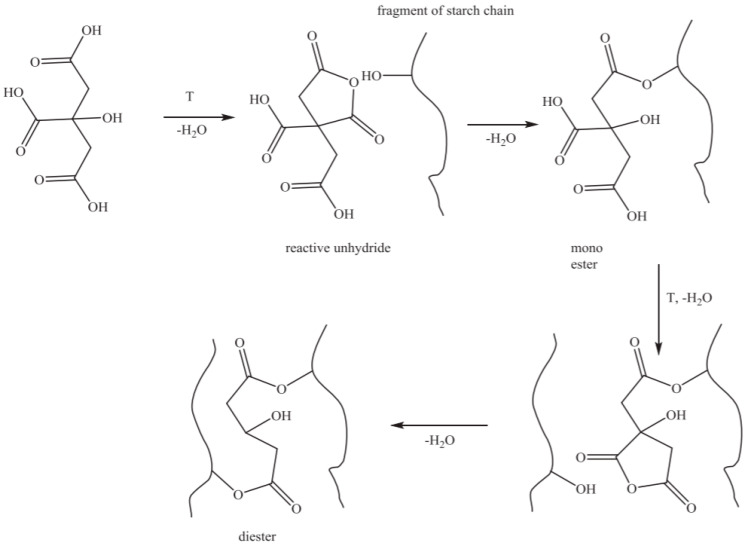
The mechanism of starch esterification by citric acid Reprinted/adapted with permission from Ref. [12]. Copyright © 2016, Elsevier.

Citric acid (CA) is an organic acid widely used in the food and daily chemical industries. It is non-toxic with a good solubility, anti-oxidation and mild reaction. Moreover, CA is a licensed additive and is widely used in the production of jams, juices, soft drinks, biscuits and other foods [7,8]. CA can change the composition, structure and function of starch through hydrolysis and esterification, which is mainly related to conditions, such as temperature, concentration, processing time and starch composition (amylose and amylopectin ratio) [9]. Generally speaking, when the reaction temperature is low (the temperature in the process is always lower than the starch gelatinization temperature), the hydrolysis reaction plays a major role, and at the same time, the starch undergoes a toughening reaction; when the reaction temperature is high (>100 °C), the esterification plays a major role [10,11]. As shown in Figure 2, CA contains one hydroxyl group and three carboxyl groups, which are dehydrated during heating to form citric anhydride and undergo esterification with the alcoholic hydroxyl group in the starch molecules. 

**Figure 3:**
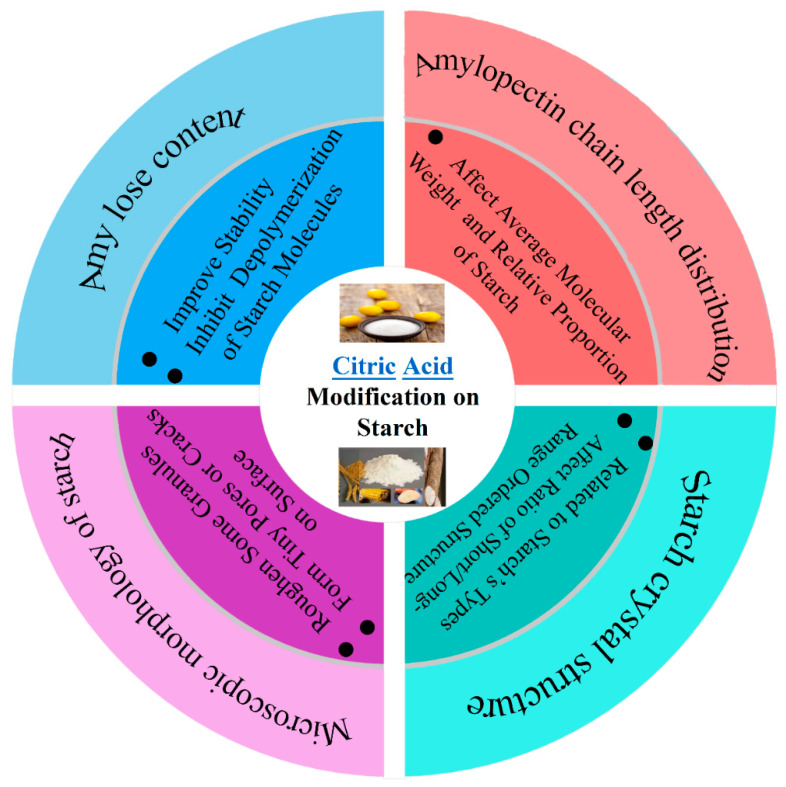
The effect of CA modification on the starch composition and structure.

**Figure 4:**
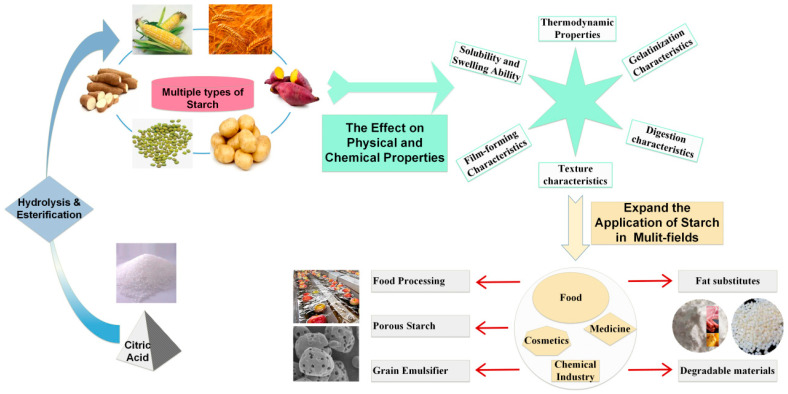
The effect of CA modification on the physical and chemical properties of starch and the application of CA-modified starch.

In this paper, the effects of CA modification on the amylose content, amylopectin chain length distribution, microscopic morphology, solubility and swelling ability, thermodynamic properties, gelatinization properties, digestibility properties, texture properties and film-forming properties in starch were described (As shown in Figure 3 and Figure 4). The application status and development trend of CA modified starch were reviewed.

## 2. The Effect of CA Modification on the Starch Composition and Structure

### 2.1. Amylose Content

The amylose content can be determined by the iodine binding method and concanavalina lectin A (ConA) sedimentation method. The iodine binding method is based on the binding capacity of amylose to iodine, and its content is determined by colorimetry. Since the long side chains of amylopectin can also bind to iodine, the assay results tend to exceed the true value. In addition, the ConA sedimentation method indirectly determines the content of amylose by enzymatic hydrolysis, based on the specific binding property of ConA and amylopectin. Since ConA binds to the less branched amylopectin moiety, its assay results also exceed the true value [13]. In the initial stage of the HCl hydrolysis of starch, starch granules were depolymerized and the content of amylose decreased significantly, which was mainly related to the different degrees of tolerance of the amorphous and crystalline regions of the starch granules to HCl. Generally speaking, the amorphous region undergoes hydrolysis before the crystalline region, and acid molecules degrade or exfoliate the amorphous region, resulting in an increase in the crystallinity of the starch granules [14,15]. This conclusion is consistent with the hypothesis that Jenkins et al. proposed, that the amorphous region is mainly composed of amylose [16]. Martin et al. compared the modification effects of HCl and CA on starch and found that amylose was significantly reduced in all treatment groups. Under the same conditions, CA modification can better retain amylose, which may be related to the esterification reaction to improve the stability of amylose and inhibit the depolymerization of starch molecules [17]. Babu et al. found that with the increase of the CA concentration and the prolongation of the hydrolysis time, the content of amylose increased significantly, which may be related to the cross-linking of amylose after hydrolysis and the depolymerization of amylopectin [18,19].

### 2.2. Amylopectin Chain Length Distribution

In natural starch, amylopectin exhibits a highly branched structure, and α-D-glucose is linearly connected by α-1,4 glycosidic bonds to form the main chain and each the short branch of amylopectin. The chain and each short branch are linked through α-1,6 glycosidic bonds. Generally, enzymes (isoamylase and pullulanase) are used to debranch amylopectin into short linear structures, and then high-performance size-exclusion chromatography (HPSEC) and other methods are used to separate the branched starch. The chain length distribution of chain starch was determined. Li et al. found that with the prolongation of the starch hydrolysis time by HCl, the long-chain starch and medium-chain starch in the amorphous region preferentially underwent hydrolysis, and the long-chain starch (degree of polymerization (DP) ≥ 37), medium-chain starch (DP 25–36) and short-chain starch were hydrolyzed preferentially. The relative proportions of (DP 6–24) decreased sequentially [20]. Kim et al. found that CA hydrolysis led to a slight increase in the average molecular weight of long-chain starch and medium-chain starch, and a slight decrease in the relative proportion; the average molecular weight of short-chain starch was slightly reduced, but the relative proportion increased slightly [11].

### 2.3. Microscopic Morphology of Starch

The microscopic morphology of starch from different sources includes round (wheat, sorghum starch, etc.), oval (potato, tapioca starch, etc.), polyhedron (rice, corn starch, etc.), etc. A scanning electron microscope (SEM), a light microscope (light microscopy, LM), a polarized light microscope (polarized light microscopy, PLM) and a laser confocal microscope (confocal laser scanning microscopy, CLSM) and other equipment are commonly used for detection. Huo et al. found that CA modification had no significant effect on the polyhedral structure of rice starch granules, while as the concentration increased, slight erosion marks appeared on the surface of some granules [21]. Similarly, related studies found that CA modification did not significantly change the granule morphology of other starch varieties (corn, sweet potato, yam, potato, cassava, banana and lentil, etc.). CA modification resulted in the roughening of some starch granules and the formation of tiny pores or cracks on the surface [11,17,19,22].

### 2.4. Starch Crystal Structure

Starch crystal is composed of a short-range ordered structure and long-range ordered structure. The short-range ordered structure refers to the double helical structure composed of short chain parts in amylose and amylopectin, while the long-range ordered structure refers to double helical molecules. The chains form different polymorphs in certain regions of starch granules through intermolecular interactions [23]. The short-range ordered structure can be detected by 13C CP/MAS solid-state nuclear magnetic resonance (cross-polarization magic-angle spinning nuclear magnetic resonance, 13C CP/MAS NMR), Fourier transform infrared spectroscopy (FTIR) and other methods. The C-1 (90–110 ppm) region in the 13C NMR spectrum is related to the crystalline structure of starch granules: A-type starch shows a triplet in the C-1 region, B-type starch shows a doublet in the C-1 region, The characteristic peak of the C-type starch in the C-1 region is largely determined by the relative proportions of A- and B-type starch [24]. The absorption peaks in the FTIR spectrum reflect the stretching and bending vibrations of the chemical bonds of the specific functional groups, and the ratio of (1047/1022) cm^−1^ and (1022/995) cm^−1^ peak intensities in the spectrum is regarded as an indicator of the ordered structure of the starch granules, in which the (1047/1022) cm^−1^ peak intensity ratio reflects the order degree of the starch molecules. The larger the ratio, the higher the order degree [25]. The long-range ordered structure of starch granules can be determined by X-ray diffraction (XRD), small angle X-ray scattering (SAXS), small angle neutron scattering (SANS), Raman spectrum (RS) and other methods are used for detection [26,27]. Different types of starch crystals have different characteristic of absorption diffraction peaks in the X-ray diffraction pattern, and can be divided into A-type, B-type and C-type, according to different crystal structures (a mixture of A-type and B-type) [28].

In the process of starch hydrolysis, the amorphous region degrades faster. As the acid molecules penetrate into the crystalline region, the surface layer of the crystalline region will also be degraded and the phenomenon of erosion will occur. However, the hydrolysis rate of the crystalline region is much slower than that of the amorphous region, so that the starch granules can retain the crystalline structure [29]. At present, the hypotheses about the increase of starch crystallinity caused by acid hydrolysis mainly include: (1) the amylose in starch granules undergoes hydrolysis and reorganizes to form more crystal structures [30,31]; (2) the starch microcrystalline structure forms cavities, free starch molecular chains enter the cavity to form a crystal structure [32]; (3) free amylose in starch granules recombine through hydrogen bonds to form a double helix structure [23,33]. Relevant studies have shown that with the increase of the CA concentration and the prolongation of the hydrolysis time, the crystallinity of the starch granules also shows a decreasing trend [18,21,34,35]. Huo et al. found that the hydrolysis of CA led to the shift of the scattering peaks in the X-ray diffraction pattern, and the decrease of the thickness of the semi-crystalline layer, indicating that the hydrolysis of the thin layer in the amorphous region occurred. At the same time, the CA hydrolysis leads to an increase in the proportion of ordered structures in the starch molecule, resulting in an increased crystallinity [21]. In addition, studies have shown that as the degree of CA substitution increases, the esterification reaction leads to a decrease in the crystallinity of starch granules, which may be related to the cross-linking of starch molecules in the esterification reaction, resulting in a decrease in mobility [10,17,36,37]. Moreover, the effect of esterification on starch crystallinity is also related to the type of starch. Remya et al. found that CA esterification resulted in the decrease of the crystallinity of potato starch (A-type), tapioca starch (B-type) and sweet potato starch (A-type). However, the same treatment resulted in the increase of crystallinity of lentil starch (C-type) and banana (C-type), which could be related to that C-type starch has a more tightly branched structure than A-type and B-type starches [19].

## 3. The Effect of CA Modification on the Physiochemical Properties of Starch 

### 3.1. The Solubility and Swelling Abilities of Starch 

A high temperature increases the vibration of starch molecules in water and breaks the intermolecular hydrogen bonds, resulting in more sites for starch molecules to interact with water molecules. At the same time, water molecules enter the crystallization zone, the crystalline state is transformed into an amorphous state, and the starch undergoes irreversible swelling [38]. Most studies have shown that acid hydrolysis leads to the destruction of the network structure of starch granules and the reduction of the water holding capacity; at the same time, the hydrolysis of long-chain starch molecules into short-chain starch molecules containing a large number of hydroxyl groups leads to a decrease in the solubility of starch and an increase in the swelling capacity [4,14,39,40,41]. Similarly, related studies have shown that CA modification leads to a trend of the decreasing solubility and swelling ability of starch [10,17,35,36]. Martins et al. found that the modification of HCl and CA resulted in a decrease in the solubility and swelling ability of starch. In comparison, CA-modified starch has a lower solubility and higher swelling ability, which may react with esterification and lead to the exposed hydroxyl groups of starch granules. The decrease in number, and the polar interaction of ester bonds are related to the formation of strong hydrogen bonds [17]. In addition, Lee et al. found that the esterification reaction was pH-dependent, and as the pH increased, the CA esterification reaction weakened. When pH was 3.5 or 4.5, the swelling ability of modified starch decreased significantly; when pH was 5.5, the swelling ability of modified starch was close to that of native starch [35].

### 3.2. Thermodynamic Properties of Starch 

The thermodynamic properties of starch can be measured by differential scanning calorimetry (DSC) through the initial gelatinization temperature (T_0_), peak gelatinization temperature (T_C_), termination gelatinization temperature (T_P_) and gelatinization enthalpy (ΔH) characterized in the DSC curve. It is generally believed that starch hydrolysis results in the destruction of the amorphous region, requiring more energy to melt the crystalline structure during the heating process, and increasing ΔH [14,40,42]. At the same time, amylose and amylopectin are degraded to varying degrees and the degree of branching is reduced, resulting in a decrease in the molecular migration resistance, which is favorable for the orientation rearrangement, and the degree of retrogradation of starch increases [14,40]. However, the gelatinization temperature of hydrolyzed starch showed different trends. Some studies showed that starch hydrolysis led to an increase in the gelatinization temperature [14,43,44], while other studies showed that the gelatinization temperature of starch decreased [40,45], mainly related to the hydrolysis of the amorphous region and the degradation level of amylose and amylopectin [46]. Relevant studies have shown that CA modification leads to an increase in the starch gelatinization temperature, a decrease in ΔH, and a decrease in retrogradation. On the one hand, the process of starch hydrolysis is also a toughening process. The toughening treatment leads to the greater mobility of starch molecules, the enhanced interaction between the starch molecular chains, and the increase of the gelatinization temperature. On the other hand, esterification leads to the cross-linking of starch molecular chains, increasing the orientation of the amorphous region, and inhibiting the reorganization of starch molecular chains [10,11,36]. However, studies have also shown that CA modification leads to an increase in starch ΔH, which may lead to the formation of double helical structures and amylose-lipid complexes in starch molecules through esterification [34].

### 3.3. Starch Gelatinization Characteristics

Starch gelatinization characteristics directly affect the processing characteristics and sensory quality of starch-based foods. Generally, a rapid viscosity analyzer (RVA) is used to measure, through the peak viscosity (peak viscosity), valley value in the RVA curve trough viscosity, holding strength, final viscosity, break down viscosity, setback, peak time and pasting temperature and other indicators to characterize. Studies have found that starch hydrolysis leads to a significant reduction in gelatinization viscosity, which is mainly related to the destruction of the amorphous region and the reduction of the solubility and swelling ability of starch granules [40,45,47]. Likewise, CA modification resulted in a decrease in peak viscosity, valley viscosity and final viscosity of starch, while there was an increase in the disintegration value. The disintegration value is the difference between the peak viscosity and the valley viscosity. The increase of the disintegration value indicates that the shear resistance of the starch is deteriorated, which may promote the cross-linking of the starch molecular chain with the esterification reaction, which inhibits the regeneration of the amylose molecules during the cooling process [17,18].

### 3.4. Digestion Characteristics of Starch

According to the digestibility, starch can be divided into rapidly digestible starch (RDS), slowly digestible starch (SDS) and resistant starch (RS). Fast-digestible starch refers to starch that is rapidly digested and absorbed in the small intestine (<20 min). Slow-digestible starch refers to starch that is completely digested and absorbed in the small intestine but at a slower rate (20–120 min). Resistant starch refers to starch that cannot be digested and absorbed in the small intestine, but can be partially fermented in the large intestine by intestinal microorganisms [48]. The digestibility of starch is related to its source, particle size, ratio of amylose to amylopectin, amylose-lipid complex, amylose content, amylopectin chain length distribution, crystallinity, crystal type and other factors [49]. Relevant studies showed that hydrolysis had no significant effect on the digestibility of gelatinized starch, and the contents of RDS, SDS and RS did not change significantly. During the cooking process, the crystal structure of starch granules is destroyed, water molecules enter the crystallization zone, and the crystalline state is transformed into an amorphous state [50,51,52]. Miao et al. found that hydrolysis resulted in a significant change in the digestive properties of native starch, with an increase in the RDS content and a decrease in the SDS and RS contents. With the extension of hydrolysis time, the content of RS showed a gradually increasing trend. In the initial stage of starch hydrolysis, starch granules are highly sensitive to amylase, and the amorphous region is mainly destroyed, and the effective area of amylase binding and hydrolysis increases; as the degree of hydrolysis gradually increases, the crystallinity of starch also increases. Sensitivity to amylase gradually decreases [53]. Related studies have found that CA modification leads to a decrease in the content of RDS and an increase in the content of SDS and RS in starch [10,19,35,36,54,55]. Shaikh et al. found that after CA treatment of natural corn starch, the RDS content decreased from 22.77% to 7.63%, the SDS content decreased from 12.74% to 4.41% and the RS content increased from 64.66% to 87.96%. Navaf found that the in-vitro digestibility of talipot palm starch was decreased by citric acid treatment, and that of the slowly digestible starch (SDS) and resistant starches (RSs) increased significantly (*p* ≤ 0.05) from 31.71% to 39.43% and 37.55% to 53.38%, respectively [56]. On the one hand, the hydrolysis reaction leads to a decrease in the molecular weight of starch chains, and some short-chain starch molecules form a double helix structure that resists enzymatic hydrolysis through hydrogen bonds; on the other hand, the esterification reaction promotes the cross-linking of starch chains, making starch resistant to enzymatic hydrolysis [10].

### 3.5. Texture Characteristics of Starch 

The texture characteristics of starch can be detected by a texture profile analysis (TPA), using a physical property analyzer. Parameters, such as gumminess, springiness, and chewiness are characterized. Studies have found that with the increase of the degree of hydrolysis, the gel strength of starch gradually decreases, which is mainly related to the depolymerization of starch granules and the degradation of amylose molecules [44,45]. Moin et al. found that HCl hydrolysis resulted in a significant decrease in the hardness, chewiness, and cohesiveness of starch gels, and a gradual increase in gel strength during refrigeration [45]. Similarly, the gel strength of starch also showed a tendency to decrease due to CA modification [10,18,57,58]. Babu et al. found that with the increase of CA concentration and the prolongation of the hydrolysis time, the hardness and chewiness of starch gel gradually decreased, and the adhesive force gradually increased, but the elasticity did not change significantly. On the one hand, the hydrolysis reaction leads to the decrease of long-chain starch molecules and the increase of short-chain starch molecules; on the other hand, the esterification reaction leads to the cross-linking of starch molecules, which reduces the mobility of starch chain molecules, which is not conducive to the reorganization of starch molecular chains [59].

### 3.6. Starch Film-Forming Characteristics

Utilizing its retrogradation property, starch can be made into films through gelatinization, cooling, retrogradation, drying and other processes. Starch film has the characteristics of a good stretchability, transparency, folding resistance, water insolubility and low air permeability, which can be applied in the field of green packaging. Studies have shown that the hydrolysis reaction can improve the mechanical strength and water vapor transmission rate of starch films and reduce the elongation at break. Retrogradation of starch is mainly related to the binding of amylose molecules. Amylopectin molecules are not prone to retrogradation because of the branched structure, which have an inhibitory effect on the retrogradation of amylose. The hydrolysis reaction leads to a decrease in the molecular weight of amylopectin in starch granules and a decrease in the degree of branching. In the film forming solution, the small molecular weight starch molecular chains are dispersed in the gaps of the amylopectin molecules, which are conducive to the formation of an orderly arrangement of the starch molecular chains through hydrogen bonding during the film forming process. The water vapor transmission rate is mainly related to the polar groups in the starch film. The starch molecular chain produced by hydrolysis has a large number of hydroxyl groups, which can improve the dissolution and diffusion of water vapor in the starch film [47]. Similarly, studies have found that CA modification can improve the mechanical strength and thermodynamic stability of starch films and reduce the water vapor transmission rate, which is mainly related to the cross-linking between starch molecules caused by the esterification reaction and the strengthening of the interaction between starch molecules [7,60,61].

## 4. The Application of CA-Modified Starch

### 4.1. Food Processing

CA has the characteristics of a good solubility, pure acidity, strong chelating ability and low toxicity, and has been certified by the US FDA as a “generally recognized as safe” (GRAS) food additive, can be used in food processing as a sour agent, antioxidant, buffer, color retention agent, etc. Rice is one of the most commonly consumed staple food around the world and is an excellent source of starch. Cooked rice generally has a high blood sugar response, which is not good for the blood sugar control of diabetic patients [62]. Lee et al. soaked rice in a CA solution for 24 h, and then dried it for frying or puffing, and found that this method could help improve the digestive properties of rice. In the process of high temperature and high pressure, the esterification reaction leads to the cross-linking of starch molecules, and the surface structure of cooked rice is more compact and complete, thereby inhibiting the hydrolysis of amylase. At the same time, the content of RS increased and the content of RDS decreased in the samples pretreated with CA, which improved the satiety of rice and slowed down the digestion rate [61]. Liu et al. found that the hydrothermal treatment (HMT)- (80 °C) and CA- (0.07–0.35%) modified PP, effectively enhanced the dough strength against mixing and suppressed retrogradation, which showed that hydrothermal-acidic treatment is an effective way to overcome dough mixing and retrogradation shortages in the potato pulp-based staple production [63].

### 4.2. Porous Starch

Porous starch is a modified starch, which can be used as a catalyst, adsorbent, slow-release agent, etc. in different fields, and has the advantages of a high efficiency, non-toxicity, safety, and biodegradability. Porous starch can be obtained by hydrolyzing starch with enzymes or acids below the gelatinization temperature. The use of CA as a hydrolysis catalyst has the advantages of safety and controllability, low price, and mild hydrolysis. Kim et al. found that the hydrolysis of CA resulted in the partial breakage of the crystal structure of corn starch and an increase in the area of the central cavity of starch granules, resulting in a significant increase in its adsorption capacity in water and oil [11]. Similarly, Qi Ruonan et al. found that the hydrolysis of CA led to an increase in the number of pores on the surface of starch granules, a larger internal space, and a significant increase in the adsorption of tartrazine.

### 4.3. Grain Emulsifier

Pickering emulsion is a stable emulsion that replaces traditional surfactants with solid particles, which has important application value in the fields of food, medicine and cosmetics. Ordinary starch has a large particle size (>10 μm) and weak hydrophobicity, making it difficult to adsorb on the oil-water interface to form a stable emulsion. Therefore, CA treatment can improve the hydrophobicity of starch, preparing a good starch based solid particle emulsifier. Pickering emulsion, prepared with CA modified starch as emulsifier has a good emulsifying ability and storage stability. The diameter of oil droplets in the emulsion is small and uniform, and the oil droplets are not easy to coalesce. The emulsion prepared from natural starch depolymerized after being stored at 25 ℃ for 3 h, while the emulsion prepared from CA modified starch still maintained a good stability under the same conditions for 12 h, and its emulsification index reached more than 90%. The esterification reaction can improve the hydrophobicity of starch molecules and reduce the surface tension of the oil-water interface [37]. Lv et al. also found that esterified tigernut starch with citric acid possess excellent Pickering emulsifying ability [64].

### 4.4. Fat Substitutes

Starch-based fat substitutes have texture, taste and stability similar to those of fat-containing foods, and are recognized as safe fat substitutes, which have attracted more and more attention. However, most natural starches have the characteristics of easy aging, a low shear resistance, and poor thermal stability, and are not suitable as fat substitutes. CA modified starch has been prepared as starch-based fat substitutes in different food matrices, such as mayonnaise. Park et al. compared the physicochemical properties of octenyl succinic anhydride modified starch, toughened modified starch, CA modified starch, acetylated modified starch and moist heat modified starch, and found that CA modification can improve the solubility, water holding capacity and swelling capacity of starch. Moreover, the mayonnaise product obtained by replacing oil and fat with CA-modified starch has a good color, viscoelasticity and emulsion stability [65].

### 4.5. Degradable Materials

Starch-based biodegradable materials have an excellent biocompatibility and biodegradability, and can be used in packaging, construction, medicine and other fields. Starch-based film materials have the characteristics of being colorless, odorless, good flexibility, high transparency and impermeability to oxygen, etc., but also have disadvantages, such as poor mechanical properties and poor water vapor permeability. Related studies have found that CA modification can improve the mechanical strength, barrier properties, tensile strength and thermal stability of starch films, and reduce the water vapor transmission rate, which endowed its application in the bioactive packaging field [7,66]. Likewise, starch-based foams prepared with CA-modified starch as the substrate have a good flexural strength, thermal stability, and water resistance [67,68]. Moreover, CA-modified starch also has potential application value in the preparation of disintegrants and tissue engineering scaffolds. Pachuau et al. found that using CA-modified taro starch as raw materials to prepare disintegrants can increase its dissolution efficiency and reduce the average dissolution time, thereby improve the drug bioavailability [69]. In addition, Kavousi et al. used CA-modified starch to prepare oil in water (O/W) medium internal phase emulsion (MIPE) to synthesize polymer interconnected porous materials. The material has a good cytocompatibility and can be used as a tissue engineering bioscaffold for cell culture [70]. Kalita et al. reported that citrated starch from the glutinous Assam bora rice exhibited a better packing rearrangement and cohesive properties than standard corn starch, which are potentially functional and sustainable materials for pharmaceutical industries [71].

## 5. Prospects

CA treatment is an convenient, mild and environmentally friendly strategy to modify the composition, structure and function of starch through hydrolysis and esterification, which expands the application of starch in the fields of food, cosmetics, chemical industry and medicine. In recent years, scholars have made progress in the study of modified starch with CA, while there are still many challenges in the maturity and commercial application of this technology. First of all, the mechanism of CA modification on the crystal structure of starch granules is not yet clear. The changes in the degradation of the amorphous region, the depolymerization and repolymerization of starch molecules, and the formation of amylose-lipid complexes during the starch modification process can be further explored. Secondly, in order to deeply understand the mechanism of CA-modified starch on the nutritional quality and texture properties of food, the interaction of esterified-modified starch with protein, dietary fiber, fat and other components in a high temperature and high pressure environment need to be investigated. Thirdly, in order to further improve the physiochemical properties and producing efficiency of CA-modified starch, CA modification can be combined with other methods, such as enzymatic hydrolysis, extrusion, microwave, ultrasonication and radiation [72,73]. Finally, starch as a natural polymer, still suffers from a poor thermal stability and easy aging. The starch-based composites prepared from CA-modified starch have a good water resistance, heat resistance, mechanical strength and different degradation abilities which have a good application prospect.

## Data Availability

The data presented in this study are available on request from the corresponding author.

## Abbreviations

CA: citric Acid; DP: degree of polymerization; O/W: oil in water; RDS: ready digestible starch; RS: resistant starch; SDS: slowly digestible starch.
